# Receptor binding domain-independent pancoronavirus vaccine design by fusion of conserved T/B Epitopes

**DOI:** 10.1080/22221751.2026.2631206

**Published:** 2026-02-11

**Authors:** Yunru Yang, Yetian Chen, Mengyu Hong, Ronghua Zou, Jingxue Yao, Entao Li, Jiayi Wang, Xiaodong Ye, Yixiang Xing, Yangming Tang, Xiaojie Lu, Chengchao Ding, Hongliang He, Dali Tong, Yuhua Shang, Jian Wang, Guangyu Zhao, Xiaoxue Huang, Fuli Feng, Qingyu Cheng, Bofeng Li, Baoying Huang, Wenjie Tan, Sandra Chiu, Tengchuan Jin

**Affiliations:** a Department of Infectious Diseases, The First Affiliated Hospital of USTC, Division of Life Sciences and Medicine, Center for Advanced Interdisciplinary Science and Biomedicine of IHM, University of Science and Technology of China, Hefei, People’s Republic of China; b Laboratory of Structural Immunology, State Key Laboratory of Immune Response and Immunotherapy, Division of Life Sciences and Medicine, University of Science and Technology of China, Hefei, People’s Republic of China; c Anhui Genebiol Biotech. Ltd., Hefei, People’s Republic of China; d Institute of Health and Medicine, Hefei Comprehensive National Science Center, Hefei, People’s Republic of China; e Division of Life Sciences and Medicine, University of Science and Technology of China, Hefei, People’s Republic of China; f Department of Chemical Physics, University of Science and Technology of China, Hefei, People’s Republic of China; g Department of Pathology, Tongling People's Hospital, Tongling, People’s Republic of China; h Hybio Pharmaceutical Co. Ltd., Shenzhen, People’s Republic of China; i School of Artificial Intelligence and Data Science, University of Science & Technology of China, Hefei, People’s Republic of China; j Department of Laboratory Medicine, The First Affiliated Hospital of USTC, Division of Life Sciences and Medicine, University of Science and Technology of China, Hefei, People’s Republic of China; k State Key Laboratory of Pathogen and Biosecurity, Academy of Military Medical Sciences, Beijing, People’s Republic of China; l School of Artificial Intelligence and Data Science, University of Science & Technology of China; m National Institute for Viral Disease Control and Prevention, People’s Republic of China CDC, Beijing, People’s Republic of China; n Key Laboratory of Anhui Province for Emerging and Reemerging Infectious Diseases, Hefei, People’s Republic of China; o Biomedical Sciences and Health Laboratory of Anhui Province, University of Science & Technology of China, Hefei, People’s Republic of China; p Clinical Research Hospital of Chinese Academy of Sciences, University of Science and Technology of China, Hefei, People’s Republic of China

**Keywords:** Pancoronavirus, vaccine, epitopes, fusion protein, cross-protective

## Abstract

The persistent emergence of SARS-CoV-2 variants continues to compromise current vaccine efficacy, driving the development of broad-spectrum coronavirus vaccines to address variant evasion and future outbreaks. To develop a pan-coronavirus vaccine, we identified some conserved T/B epitopes across spike proteins of human-infecting coronaviruses, focusing on two conserved long peptides, VV and VS, which demonstrated broad immunogenicity in PBMCs from COVID-19 convalescent patients. By structurally fusing the VV and VS long peptides with heptad repeat 1/2 (HR1/2) domains from the S2 subunit, we engineered a trimeric immunogen HR1-VV-HR2-VS. This design induced superior cellular and humoral immune responses compared to individual peptide components in immunized mice. The vaccine also significantly reduced viral loads and attenuated lung pathology in mice challenged with HCoV-229E, SARS-CoV-2 prototype strain, and the KP.2 variant, demonstrating cross-protective immunity. Therefore, these results indicated that HR1-VV-HR2-VS vaccine elicits cross-protective immunity, highlighting its potential as a universal coronavirus vaccine. In addition, we developed an innovative peptide vaccine platform based on the HR1-HR2 trimeric structural protein, which serves as a potent polypeptide fusion scaffold to significantly enhance peptide immunogenicity.

## Introduction

WHO has declared SARS-CoV-2 no longer a Public Health Emergency of International Concern, yet persistent viral circulation and evolution create ongoing epidemiological uncertainty. Although early vaccines primarily targeted immunodominant regions of the spike (S) protein, particularly the receptor-binding domain (RBD) [1–5], the accumulation of mutations in these regions has facilitated immune evasion [6–9], thereby diminishing the efficacy of prototype-strain-based vaccines [10–16]. Consequently, developing pan-coronavirus vaccines against conserved epitopes remains crucial for outbreak control.

Current design strategies for broad-spectrum coronavirus vaccines primarily focus on two approaches: (i) targeting conserved S protein domains (e.g. HR121 and HR1LS vaccines), and (ii) multi-epitope vaccines designed to elicit synergistic B-cell and T-cell responses (e.g. UB-612 and RBD-HLA-EP vaccines) [17–20]. While conserved epitope-based vaccines elicited potent neutralizing antibody responses, their long-term efficacy is fundamentally constrained by the transient nature of humoral immunity. In contrast, combinatorial vaccines enhance cross-variant protection by integrating RBD-directed antibodies with conserved T-cell epitopes. However, RBD-focused approaches may accelerate immune escape, necessitating strategies targeting more stable viral regions. Thus, the synergistic action of conserved B cell and T cell epitopes in next-generation vaccines may overcome current limitations.

Developing effective T/B cell epitope vaccines faces main two challenges: HLA restriction and suboptimal immunogenicity. Studies on HPV vaccines have showed that synthetic long peptides (SLPs) of the E7 antigen induce more potent antigen-specific CD8^+^ T cell responses than minimal epitope peptides [21]. More importantly, long peptide designs enable the integration of multiple epitopes, thereby broadening HLA coverage. Multiple COVID-19 vaccine studies have demonstrated that oligomerization of the RBD protein significantly enhances its immunogenicity [22–24]. Building on these principles, we designed a unique vaccine platform that structurally integrates conserved T/B cell epitopes into a heptad repeat 1-heptad repeat 2 (HR1-HR2) trimeric scaffold protein. This multivalent approach synergistically combines the broad immune recognition of SLPs, offering a promising strategy for developing a potent pan-coronavirus vaccine.

## Materials and methods

### Animals, human samples and viruses

Specific pathogen-free female C57BL/6JGpt (N000013), BALB/cJGpt (N000020), K18-hACE2 (T037657), and HLA-A*02:01 transgenic mice were obtained from GemPharmatech and CAMS & PUMC. All procedures were approved by USTC (USTCACUC24060123027). COVID-19 patient PBMCs were collected from the First Affiliated Hospital of USTC (2023-ky-001). HCoV-229E, SARS-CoV-2 (Wuhan-Hu-1), and KP.2 were prepared at USTC ABSL-3. HCoV-229E challenges (IHM-AP-2024-075) were conducted at ABSL-2, while SARS-CoV-2 challenges were performed at ABSL-3 facilities.

### Peptides and adjuvants

Peptides (>95% purity) were synthesized by Hybio Pharmaceutical, with VS peptide dissolved in PBS/10% DMSO and others in PBS. Adjuvants included CpG/Al(OH)3 (Zhuoyi Biological), Montanide™ ISA 720 VG (Hybio), and Freund's incomplete adjuvant (Sigma, F5506). Inactivated vaccine was provided by Shenzhen Kangtai Biological.

### Plasmid and cells

The pcDNA3.1-NL63-S-ΔC18 expression plasmid was synthesized by General Biosystems (Anhui) Co., Ltd. The pN-1-eGFP plasmid was obtained from our laboratory. The HEK293 T and HEK293T-hACE2 cell lines were provided by Prof. Xue Tian's laboratory at the USTC. Huh-7 cells were purchased from Wuhan Pricella Biotechnology Co., Ltd. The HEK293 T, HEK293T-hACE2, N2A, RAW264.7 and Huh-7 cell lines were cultured in Dulbecco’s modified Eagle’s medium (DMEM, Servicebio) supplemented with 10% fetal bovine serum (FBS, Servicebio). The medium for the HEK293F cell line was purchased from Sino Biological (M293CD1).

### Expression and purification of the fusion protein

The SARS-CoV-2 HR1 (residues 910-974) and HR2 (residues 1156-1203) domains (MT019533.1) were fused via a SGGRGG linker to generate HR1-HR2 [25,26]. The HR1-VV-HR2-VS construct was created by inserting VV and VS peptides at the C-termini of HR1 and HR2, respectively. These constructs were cloned into PTT5 plasmids containing secretory signals, Fc tags, and TEV cleavage sites. For protein production, HEK293F cells were transfected with the constructs using PEI and cultured (37°C, 5% CO_2_, 120 rpm) for 4 days. The supernatant was collected by centrifugation (5000 rpm, 10 min), and the Fc-fusion proteins were purified by Protein A affinity chromatography (elution with 0.1 M acetic acid). Following TEV protease cleavage to remove Fc tags, the target proteins were further purified by size-exclusion chromatography (Superdex™ 200) and analysed by SDS-PAGE. Protein deglycosylation was performed using the PNGase F Kit (Beyotime, P2318S), and the results were analysed by SDS-PAGE.

### Protein cross-linking

Protein cross-linking was performed by incubating 4 nmol of either HR1-HR2 or HR1-VV-HR2-VS fusion protein in 50 μL of 1 × PBS buffer with 2 mM disuccinimidyl suberate (DSS) at 25°C for 30 min. The reaction was terminated by adding Tris-HCl (pH 7.5) to a final concentration of 50 mM. The samples were then mixed with SDS loading buffer, denatured at 100°C for 10 min, and subsequently analysed by SDS-PAGE followed by staining and imaging.

### Circular dichroism

HR1-HR2 and HR1-VV-HR2-VS were diluted to 0.5 mg/mL with 1 × PBS and then analysed via a Chirascan qCD spectropolarimeter (Applied Photophysics). The temperature was increased from 20°C to 95°C, and the samples were scanned (180-300 nm) every 5°C. The negative control consisted of 1 × PBS. Finally, the results were analysed via GraphPad Prism.

### Analytical ultracentrifugation

Protein oligomerization states were assessed following Ni et al. [27] HR1-VV-HR2-VS and HR1-HR2 proteins (0.5 mg/mL in PBS) were analysed using a Beckman Coulter Proteomelab XL-A ultracentrifuge with an An-60 Ti rotor. After temperature equilibration (20°C, 2.5 h), samples were centrifuged at 56,000 rpm with 200 absorbance scans (280 nm) recorded at 3-min intervals. Data were processed using SEDFIT's c(s) model [28], with buffer parameters calculated by SEDNTERP for sedimentation coefficient and molecular weight determination [29].

### Enzyme-linked immunosorbent assay

Blood samples were collected 14 days post-immunization and processed for serum isolation (5000 rpm, 5 min). ELISA was performed as previously described [30] with 0.2 µg/well coating protein or 1 µg/well peptide. After blocking (5% non-fat milk/PBS), serially diluted serum samples (initial 20 – or 100-fold) were incubated (1 h, RT). Following PBST washes, HRP-conjugated goat anti-mouse IgG (1:10,000; Sangon Biotech, D110087) was added. TMB substrate (Beyotime, P0209) was added for 7 min, then the reaction terminated with 1 M H_2_SO_4_ and the absorbance was measured at 450 nm.

### Cytometric bead array

The frozen peripheral blood mononuclear cells (PBMCs) from COVID-19 convalescent patients were resuspended and washed in RPMI 1640 medium (Gibco). The cells were equally distributed into 3 wells of a 96-well plate containing complete RPMI 1640 medium supplemented with 20 μg/mL VV, 20 μg/mL VS, and 0.5% DMSO. Alternatively, the cells were added to three wells of a 96-well plate containing 5 μg/mL each VV peptide (SP1, SP2, SP3, SP4, or SP5), 5 μg/mL each VS peptide (SP6 or SP7), or 1 × PBS. The cells were cultured at 5% CO_2_ and 37°C for 24 h. The supernatant was detected via a human Th1/Th2 Cytokine CBA Kit (550749, BD Biosciences) and analysed via flow cytometry (CytoFLEX, Beckman Coulter). The results were analysed via FCAP software.

### Activation-induced marker assay

PBMCs from COVID-19 convalescents were cultured in RPMI 1640 complete medium (Gibco) with peptide stimuli (20 μg/mL VV, 20 μg/mL VS, 5 μg/mL each of the short VV peptides (SP1, SP2, SP3, SP4, and SP5), 5 μg/mL each of the short VS peptides (SP6 and SP7)) for 24 h (37°C, 5% CO_2_). Cells were stained with Zombie Red™ viability dye (BioLegend, 423109) and the following antibody cocktail (1 h, ice, dark): anti-CD4-APC/Cy7 (300518), anti-CD8-PE/Cy7 (344750), anti-CD25-FITC (985812), anti-OX40-BV421 (350013), anti-CD69-BV510 (310935), anti-CCR7-APC (353213), and anti-CD45RA-PE (304108; all BioLegend). Flow cytometry was performed on a CytoFLEX (Beckman Coulter) and analysed using FlowJo v10.

### Mouse immunization

Mice (6-8 weeks old) received three intraperitoneal immunizations (weeks 0/2/4) with either 20 μg recombinant protein or 50 μg long peptide adjuvanted with 20 μg CpG and 100 μg Al(OH)_3_. Alternatively, the first dose was administered subcutaneously with IFA/CpG adjuvants while maintaining subsequent doses.

For challenge studies, Balb/c and K18-hACE2 mice received prime-boost immunization: prime with inactivated vaccine followed by two 20 μg recombinant protein boosts adjuvanted with either Montanide ISA 720VG or CpG/Alum.

### Flow cytometry

Fourteen days post-immunization, splenocytes were isolated from euthanized mice and plated at 1-2 × 10^6 cells/well in 96-well U-bottom plates. Cells were stained with Zombie Red™ Fixable Viability Kit (423109) and blocked with an anti-mouse CD16/CD32 monoclonal antibody (101302). The cells were subsequently stained on ice in the dark for 1 h with anti-CD3-PerCP/Cy5.5 (100328), anti-CD4-APC/Cy7 (100525), anti-CD4-FITC (100406), anti-CD8-BV510 (100752), anti-PD-1-PE/Cy7 (109110), anti-CXCR5-PE (145503), anti-CD19-APC/Cy7 (152411), anti-GL7-FITC (144619), and anti-Fas-PerCP/Cy5.5 (152609; all BioLegend) antibodies. Samples were analysed on a CytoFLEX flow cytometer (Beckman Coulter) using FlowJo v10.

### Intracellular cytokine staining

Splenocytes were cultured in anti-CD28 antibodies (1 μg/mL, Biolegend 102101) RPMI 1640 medium with peptide (20 μg/mL) or protein (5 μg/mL) stimuli (PBS control) for 24 h (37°C, 5% CO2). Protein transport inhibitors (monensin & brefeldin A, Biolegend 420701/420601) were added at 18 h. Cells were then stained with the Zombie Red™ Fixable Viability Kit and anti-mouse CD16/CD32 monoclonal antibody. The cells were subsequently stained on ice in the dark for 1 h with anti-CD45-APC/Cy7 antibodies (103116), anti-CD4-FITC antibodies (100406), and anti-CD8-PerCP/Cy5.5 antibodies (100734; all Biolegend). Afterward, the cells were fixed and permeabilized via a kit (554714, BD Biosciences). The cells were subsequently stained with BV421-conjugated anti-IFN-γ antibodies (505830, BioLegend) and incubated on ice in the dark for 1 h. Samples were analysed by CytoFLEX (Beckman Coulter) using FlowJo v10.

### Cell fusion inhibition assays

HEK293 T cells (70-90% confluent) were co-transfected with spike plasmids (pcDNA3.1-SARS-CoV-2-S, – Omicron-S, or – NL63-S-ΔC18) and pN-1-eGFP using Lipo8000 (Beyotime C0533), with pN-1-eGFP alone as control. In accordance with published methods,[31] a truncated protein with an 18 amino acid deletion at the C-terminus of the NL63 spike protein was used. After 36 h, transfected cells were digested, diluted to 10^4^ cells/well, and pre-incubated with serum (10% dilution) for 1 h (37°C, 5% CO2). HEK293T-hACE2 cells (1.5 × 10^4^/well) were then added. Following 6 h co-culture, fluorescence was quantified by microscopy (5 fields/well).

### Pseudovirus neutralization experiment

Following Ding et al. [32], HEK293 T cells in 10 cm dishes (70-90% confluency) were co-transfected with pNL4-3 Luc-R-E and spike plasmids (pcDNA3.1-SARS-CoV-2-S/-Omicron-S/-NL63-S-ΔC18) using Lipo8000 (Beyotime C0533). Pseudovirus-containing supernatants were harvested after 48 h. Pseudoviruses containing 5 μg/mL polybrene (40804ES76, Yeasen) were mixed with various dilutions of serum and then incubated at 37°C in a 5% CO_2_ incubator for 1 h. Then, 1.5 × 10^4^/well HEK293T-hACE2 cells were transferred to each well of a 96-well plate that had been cultured one day in advance. Following 48-hour co-culture, supernatants were replaced with PBS and Britelite Plus reagent (PerkinElmer). Luminescence was measured using a PE Ensight instrument.

### Antibody-dependent cellular cytotoxicity

The antibody-dependent cellular cytotoxicity (ADCC) assay was adapted from established methods [33–35]. Briefly, Neuro-2A (N2A) cells were transfected with a plasmid encoding the prototype SARS-CoV-2 spike protein using Lipo8000 transfection reagent (Beyotime, C0533). At 24 h post-transfection, cells were stained with CFSE (2 μM, Biolegend, 423801) for 5 min at 37°C and seeded at a density of 3 × 10⁴ cells per well in complete medium (DMEM supplemented with 10% FBS and 1% penicillin – streptomycin). Preliminary tests indicated that 10% serum provided the necessary sensitivity and specificity to demonstrate vaccine-induced ADCC effects in our experimental system, compared to 1% and 5% serum. Therefore, all reported ADCC data were generated using this concentration. For the assay, seeded cells were incubated with 10% mouse serum for 1 h at 37°C. Subsequently, 3 × 10⁵ splenocytes, isolated from mouse spleens using an NK cell isolation kit (Solarbio, P9310) and resuspended in complete medium, were added to each well. After 4 h of co-culture, cell viability was assessed with the Zombie Red™ Fixable Viability Kit (Biolegend, 423109). ADCC activity was calculated via the formula: (experimental group – serum-free group) / (non-specific serum group − serum-free group). Here, the non-specific serum group served as the negative control, and the serum-free group as the blank control.

### Antibody-dependent cellular phagocytosis

The antibody-dependent cellular phagocytosis (ADCP) assay was performed according to a modified version of a published protocol [36]. Briefly, N2A cells expressing the SARS-CoV-2 spike protein were prepared and stained with CFSE as described above for the ADCC assay. Following staining, 1 × 10⁵ N2A cells per well were incubated with 10% mouse serum for 1 h at 37°C. Then, 1 × 10⁵ RAW264.7 cells per well were added and co-cultured for 2 h. After co-culture, cells were stained with the Zombie Red™ Fixable Viability Kit and an anti-CD11b-BV510 antibody (Biolegend, 101263). The percentage of CFSE-positive RAW264.7 cells was quantified by flow cytometry. ADCP activity was calculated using the following formula: (experimental group – serum-free group) / (non-specific serum group – serum-free group), where the non-specific serum group served as the negative control and the serum-free group as the blank control.

### Immunization and coronavirus challenge in mice

BALB/c mice primed with inactivated vaccine (20 U/mouse) and boosted twice with HR1-VV-HR2-VS were intranasally challenged with HCoV-229E (5 × 10^4^ TCID50, two doses 24 h interval). On day 4 post-challenge, lungs were harvested for viral load analysis by qRT-PCR (Vazyme RM501/Q221) using published primers [37] and histopathological evaluation.

K18-hACE2 transgenic mice were intranasally challenged with SARS-CoV-2 prototype (5 × 10^4.5^ TCID_50_) and KP.2 (5 × 10^4.25^ TCID_50_) two weeks after the final immunization. Lung tissues were harvested on day 4 postchallenge for immunohistochemistry and viral load assays. The qRT-PCR assay was performed using the following primers targeting the ORF1ab gene: forward 5′-CCCTGTGGGTTTTACACTTAA-3′ and reverse 5′-ACGATTGTGCATCAGCTGA-3′.

### Statistics

All data are expressed as mean ± SEM. ELISA endpoint titres were determined by log10-transformed OD vs. dilution curves, with positivity defined as OD ≥ 2.1× negative controls. Statistical analyses were performed using T-test (two-group comparisons), One-way ANOVA (multi-group immunization data) and Two-way ANOVA (time-course experiments). *p < 0.05; ** *p* < 0.01; *** *p* < 0.001, **** *p* < 0.0001.

## Results

### Identification of the conserved T/B epitope regions in spike proteins

**Figure 1.**
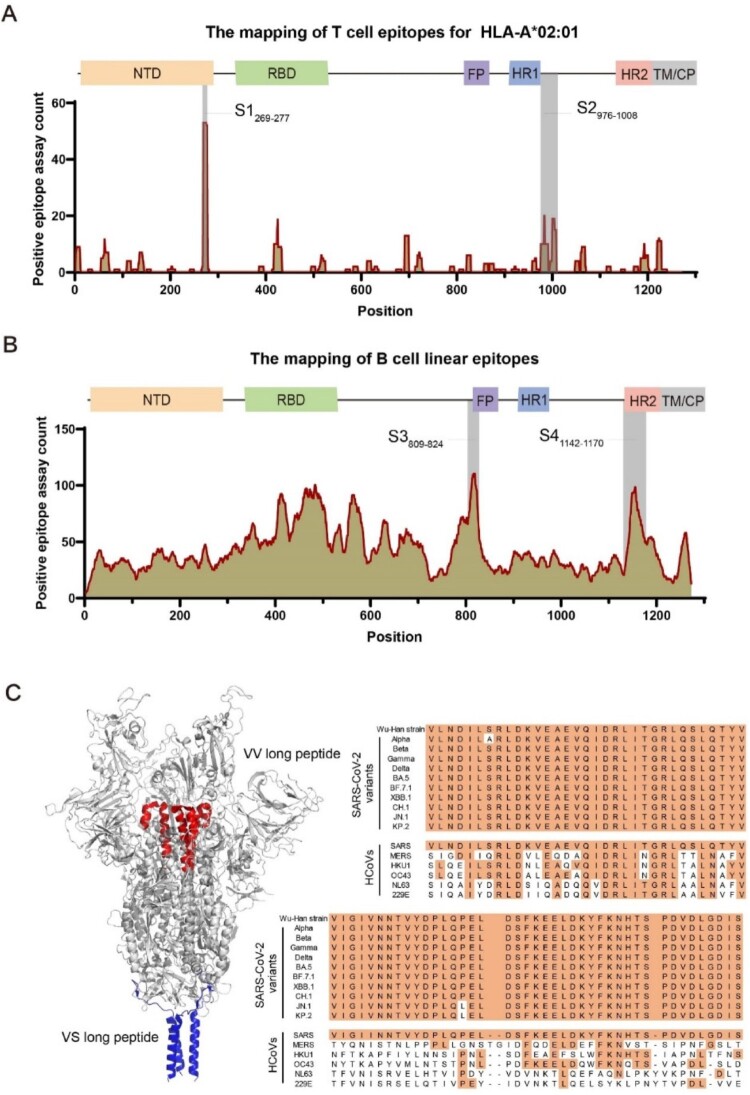
Screening of the long peptides VV and VS as candidate antigens for peptide-based vaccines. (A, B) Mapping of HLA-A*02:01-restricted SARS-CoV-2 S protein CD8^+^ T-cell epitopes (A) and linear B-cell linear epitopes (B) in the spike protein. The x-axis indicates the amino acid position in the S protein, and the y-axis represents the number of experimentally identified positive T-cell epitopes (A) or positive linear B-cell linear epitopes (B) per position. The linear schematic above depicts the corresponding domains of the S protein. Data was obtained from the IEDB database (http://tools.iedb.org/mhci/). (C) Structural localization of VV and VS long peptides in the S protein (PDB: 7VQ0). The red region corresponds to the VV peptide (residue S_976-1008_), and the blue region represents the VS peptide (residue S_1129-1170_). The right panel displayed the sequence conservation of VV and VS peptides across major SARS-CoV-2 variants (Alpha, Beta, Gamma, Delta, Omicron BA.5, BF.7.1, XBB.1, CH.1, JN.1 and KP.2) and six other human-infecting coronaviruses (SARS (NC_004718.3), MERS (KJ477103.2), HKU1 (LC315651.2), OC43 (LC315649.2), NL63 (JQ765575.1), and 229E (MT438700.1)).

The polymorphism of MHC is one of the factors limiting the protective efficacy of peptide-based vaccines. According to the Immune Epitope Database (IEDB) database, the HLA-A*02:01 allele has a high distribution frequency among HLA-A subtypes in the global population.[38] To identify the immunodominant T-cell epitopes of the SARS-CoV-2 S protein, we mapped the HLA-A*02:01-restricted CD8^+^ T-cell epitopes of the S protein on the basis of the IEDB database (Figure 1(A)). Similarly, we also mapped the B-cell linear epitopes of the S protein (Figure 1(B)). Notably, the CD8^+^ T-cell epitopes in S1_269-277_ and S2_976-1008_ presented high immunogenicity (Figure 1(A)), whereas the B-cell linear epitopes in S3_809-824_ and S4_1142-1170_ presented strong reactivity (Figure 1(B)). We subsequently performed comparative sequence analysis of these epitope regions among major circulating SARS-CoV-2 variants and six additional coronavirus species known to infect humans. Comparative sequence alignment identified the S2_976-1008_ (named as VV long peptide) and S4_1142-1170_ regions as evolutionarily conserved domains across coronavirus S proteins (Figure S1A-B). Previous studies identified that the P4 peptide (S_1139-1170_ aa) contains neutralizing antibody epitopes, and the S_1129-1145_ peptide was also confirmed to harbour B-cell antigenic epitopes [39,40]. We therefore named the extended S_1142-1170_ to S_1129-1170_ region as VS long peptide. Figure 1C presented their conservation patterns and structural positions within the S protein. IEDB analysis identified HLA-binding CD8^+^ T-cell epitopes in VV/VS regions and murine MHC-I-reactive epitopes (Figure S1C-E). The results revealed that the VV peptide contained one cross-reactive epitope, while VS contained two epitopes with dual human/murine MHC-I binding capacity (Figure S1D-E). Based on these characteristics, we synthesized five VV-derived (SP1-SP5) and two VS-derived (SP6-SP7) peptides (Table S1) for validation. Computational modelling predicted these epitopes provide 94% global population coverage (Table S2).

### Candidate long peptides induced specific T-cell immune responses in PBMCs from COVID-19 convalescent patients

**Figure 2.**
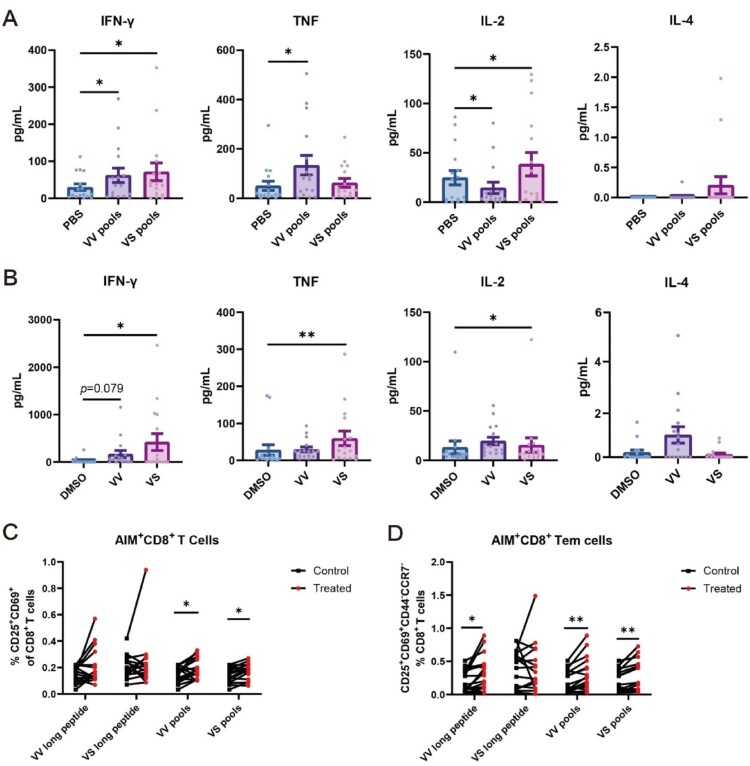
Specific T-cell immune responses of COVID-19 convalescent patients to VV/VS peptides or their peptide pools. (A) VV (SP1-5) and VS (SP6-7) peptide pools induced Th1 (IL-2/TNF/IFN-γ) and Th2 (IL-4) cytokine production in COVID-19 convalescent PBMCs (n = 16) versus PBS controls after 24 h stimulation. (B) Similar cytokine patterns were observed with VV/VS long peptides versus DMSO controls. (C, D) Proportion of AIM^+^ CD8^+^ T cells (CD8 ^+^ CD25 ^+^ CD69^+^) or AIM ^+^ CD8^+^ Tem cells (CD8 ^+^ CD45RA^-^CCR7^-^CD25 ^+^ CD69^+^) in PBMCs (n = 16) following 24 h of stimulation with VV, VS, VV pools (including SP1-5) or VS pools (including SP6-7). PBMCs stimulated with PBS or DMSO served as negative controls. A paired t test was used by A-C. *p < 0.05, **p < 0.01.

To characterize the immunogenicity of the conserved VV and VS long peptides functionally, we performed *ex vivo* stimulation assays using PBMCs isolated from 16 COVID-19 convalescent donors (Table S3). Cytometric bead array analysis showed that stimulation with the VV peptide pool significantly enhanced the secretion of IFN-γ and TNF-α, whereas the VS peptide pool predominantly boosted IFN-γ and IL-2 secretion (Figure 2(A)). Furthermore, stimulation with the VS long peptide significantly promoted coordinated secretion of Th1-type cytokines (IFN-γ, TNF-α, and IL-2; all *p* < 0.05) (Figure 2(B)). We extended our analysis to quantify activation-induced marker (AIM) positive T-cell populations in stimulated PBMCs via flow cytometry (the gating strategy was shown in Figure S2A). Quantitative analysis revealed significant expansion of both AIM ^+^ CD8^+^ T cells and AIM ^+^ CD8^+^ Tem (effector memory) cells following VS pools and VV pools stimulation (Figure 2(C,D)). Moreover, stimulation with either the VV long peptide or VS peptide pool significantly increases the proportions of both AIM ^+^ CD4^+^ T cells and AIM ^+^ CD4^+^ Tem (Figure S2B-C), confirming the robust immunostimulatory capacity of these peptides. These results provided evidence that the VV and VS long peptides could potently elicit specific T-cell immunity with broad reactivity among COVID-19 convalescent individuals.

### The peptide fusion protein HR1-VV-HR2-VS could form a trimeric conformation with highly thermal stability

**Figure 3.**
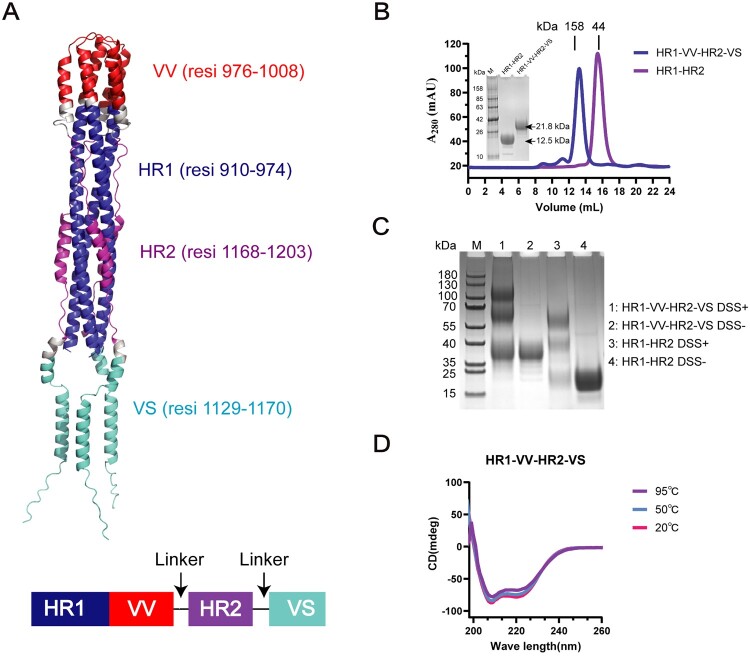
Biochemical characterization of artificially designed long peptide fusion protein HR1-VV-HR2-VS. (A) AlphaFold2 prediction of HR1-VV-HR2-VS protein suggested a homo-trimer form. The spike protein structure was shown with key regions highlighted: VV (S_976-1008_ aa, red), VS (S_1129-1170_ aa, cyan), HR1 (S_910-974_ aa, blue), and HR2 (S_1168-1203_ aa, purple). A schematic representation of the designed protein domains was provided below the structure. A flexible SGGRGG linker was introduced between the domains. (B) Purification and analysis of HR1-HR2 and HR1-VV-HR2-VS fusion proteins expressed in HEK293F cells. The purified proteins were characterized by size-exclusion chromatography and SDS-PAGE. HR1-VV-HR2-VS and HR1-HR2 proteins were indicated in blue and purple, respectively. (C) The HR1-VV-HR2-VS and HR1-HR2 recombinant proteins were crosslinked using disuccinimidyl suberate, and were further analysed via SDS-PAGE. (D) Circular dichroism spectroscopy analysis of the HR1-VV-HR2-VS protein. Scans recorded at 20°C (red), 50°C (blue), and 95°C (purple) are shown.

To enhance immunogenicity, we engineered an HR1-VV-HR2-VS fusion protein by incorporating VV and VS peptides into the conserved HR1-HR2 scaffold, a key membrane fusion domain and promising universal coronavirus vaccine target.[19, 20, 41–50] The trimeric structure was predicted using AlphaFold2 (Figure 3(A)), and both the fusion proteins were successfully expressed and purified from mammalian cells. SDS-PAGE analysis revealed that both fusion proteins migrated as discrete bands with apparent molecular masses greater than their theoretical predictions (Figure 3(B)), suggesting potential glycosylation. This hypothesis was subsequently confirmed by deglycosylation followed by SDS-PAGE (Figure S3A). This observation was corroborated by size-exclusion chromatography and analytical ultracentrifugation, which consistently measured molecular masses 30-36% higher than expected for trimeric assemblies (Figure 3(B), Figure S3B). Cross-linking analysis confirmed trimeric organization while detecting monomeric and dimeric populations (Figure 3(C)), likely due to incomplete cross-linking. Circular dichroism spectroscopy revealed predominantly α-helical structures with high thermal stability (Figure 3(D), S3C). Taken together, the above biochemical data concluded that both HR1-VV-HR2-VS and HR1-HR2 proteins adopt predominant trimeric forms in solution.

### Immunization with the fusion protein HR1-VV-HR2-VS induced markedly stronger antigen-specific T-cell immunity

**Figure 4.**
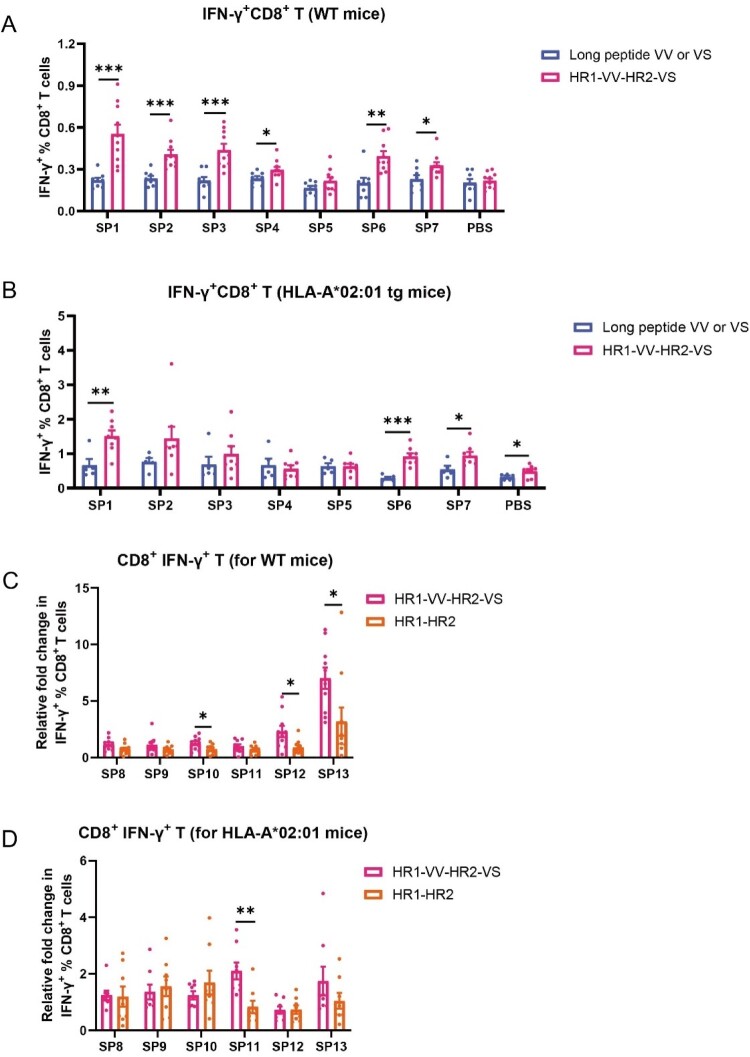
Antigen-specific T-cell immune responses induced by individual CD8^+^ T-cell epitopes in mouse splenic lymphocytes. (A, B) The percentage of IFN-γ^+^CD8^+^ T cells in splenocytes from WT mice (A) and HLA-A*02:01 transgenic mice (B) following stimulation with individual CD8^+^ T-cell epitopes. The SP1-5 peptides are located within the VV long peptide segment, while SP6 and SP7 reside in the VS long peptide region. (C-D) Fold change in the frequency of IFN-γ^+^CD8^+^ T cells among the splenocytes of wild-type (C) and HLA-A*02:01 transgenic mice (D) following single-peptide stimulation (SP8–13). The SP8-10 peptides are located within the HR1 structural domain, whereas SP11-13 peptides in the HR2 domain. Mice cohorts included: WT mice (peptide: n = 8; fusion protein: n = 10) and HLA-A*02:01 transgenics (peptide: n = 5; fusion protein: n = 8). The data were analysed by Student's t test in A-D. *p < 0.05, **p < 0.01, ***p < 0.001.

The immunogenicity of HR1-VV-HR2-VS was systematically compared to standalone VV/VS peptides in both C57BL/6 wild-type (WT) and HLA-A*02:01 transgenic mice (Figure S4A). Mice were immunized intraperitoneally (i.p.), chosen for its proven efficacy in inducing robust humoral and cellular immune protection [51–53] and to accommodate the required injection volume. Fusion protein immunization induced significantly stronger IFN-γ^+^ T cell responses against constituent epitopes (SP1-4, SP6-7) in wild-type mice compared to peptide immunization (Figure 4(A), S4B). While HLA-A*02:01 transgenic mice showed more restricted responses due to HLA limitation, enhanced IFN-γ production was still observed for specific epitopes (SP1, SP6-7), with HR1-VV-HR2-VS-immunized groups maintaining superior responses versus peptide controls (Figures 4(B), S4C). Additionally, HLA-A*02:01 transgenic mice exhibited enhanced IFN-γ^+^ T cell frequencies versus wild-type (Figures 4(A,B), S4B-C). Likewise, comparative analysis of HR1/HR2 domain epitopes (SP8-13, Table S4) revealed that HR1-VV-HR2-VS immunization enhanced IFN-γ^+^ T cell responses versus HR1-HR2 scaffold alone. WT mice showed a significantly greater fold-increase to SP10 and SP12-13 (Figures 4(C,D)), whereas SP11 responses were higher in HLA-A*02:01 transgenic mice (Figures 4(C,D)), demonstrating the dual advantage of the fusion design in augmenting both inserted and scaffold-derived epitope responses.

### Immunization with HR1-VV-HR2-VS fusion protein induced stronger germinal centre reaction

**Figure 5.**
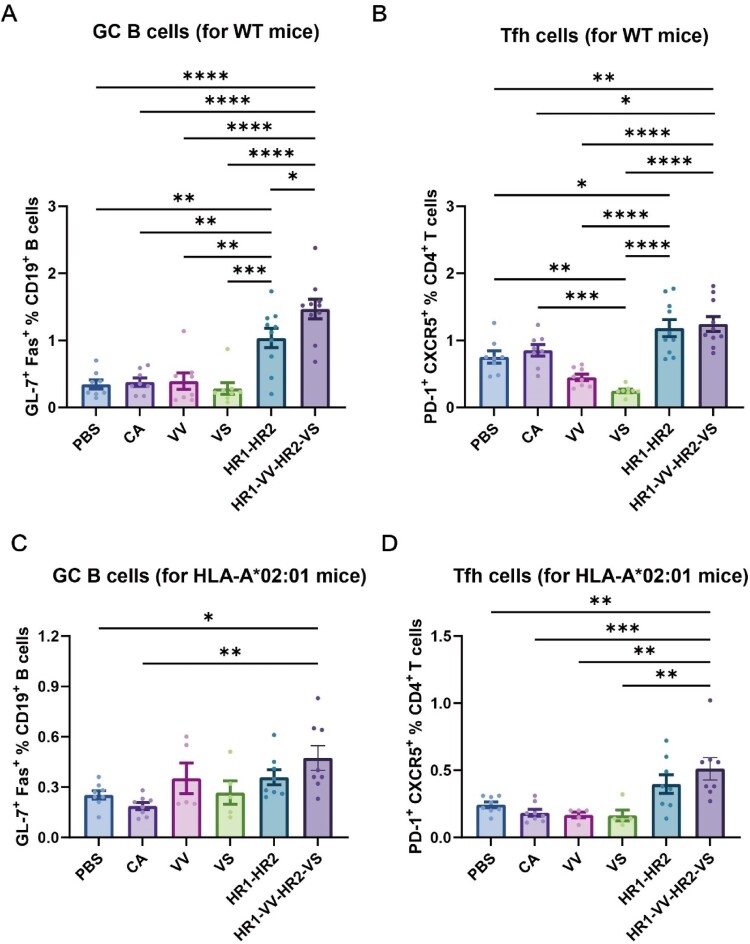
Germinal centre responses in splenocytes from immunized WT mice. (A, B) Percentages of GC B cells (CD19 ^+^ GL-7 ^+^ Fas^+^, A) and T follicular helper cells (Tfh, CD4 ^+^ CXCR5 ^+^ PD-1^+^, B) among splenocytes from WT mice. (C, D) Percentages of GC B cells (CD19 ^+^ GL-7 ^+^ Fas^+^, C) and T follicular helper cells (Tfh, CD4 ^+^ CXCR5 ^+^ PD-1^+^, D) among splenocytes from HLA-A*02:01 mice. Mice cohorts included: WT mice (PBS: n = 8; CA: CpG/Al(OH)_3_ adjuvants, n = 8; VV/VS long peptide: n = 8; HR1-HR2/ HR1-VV-HR2-VS fusion protein: n = 10) and HLA-A*02:01 transgenics (PBS/CA: n = 8; VV/VS long peptide: n = 5; HR1-HR2/ HR1-VV-HR2-VS fusion protein: n = 8). The data were analysed via one-way ANOVA. *p < 0.05, **p < 0.01, ***p < 0.001, ****p < 0.0001.

To assess antibody-mediated immunity, we quantified germinal centre (GC) B cells and T follicular helper (Tfh) cells in splenic lymphocytes from immunized WT and HLA-A*02:01 transgenic mice (gating strategy in Figure S5A). The results showed that in WT mice, the HR1-VV-HR2-VS immunization group exhibited significantly increased proportions of both GC B cells and Tfh cells compared to the PBS (GC B cells: 4.6-fold; Tfh cells: 1.7-fold), CpG/Al(OH)₃ adjuvant-only group (GC B cells: 4.1-fold; Tfh cells: 1.5-fold), and individual VV (GC B cells: 4.9-fold; Tfh cells: 2.8-fold) or VS (GC B cells: 6.2-fold; Tfh cells: 5.1-fold) long peptide immunization groups (Figure 5(A,B), Figure S5B-C). Consistent with WT mice, HLA-A*02:01 transgenic mice immunized with HR1-VV-HR2-VS demonstrated significantly elevated GC B cell (1.8-fold increase) and Tfh cell (2.0-fold increase) frequencies compared to PBS-treated controls (Figures 5(C,D), S5B-C). Of note, WT mice immunized with HR1-VV-HR2-VS indicated significantly higher frequencies of germinal centre B cells (GC B cells: 3.2-fold) and follicular helper T cells (Tfh cells: 2.5-fold) in splenocytes compared to HLA-A*02:01 transgenic mice (Figure 5). Analysis showed that although the HR1-HR2 scaffold was the primary driver of GC reactions, peptide incorporation synergistically enhanced immune responses, underscoring the superior efficacy of the fusion-based design.

### Immunization with HR1-VV-HR2-VS fusion protein induced stronger humoral immunity

**Figure 6.**
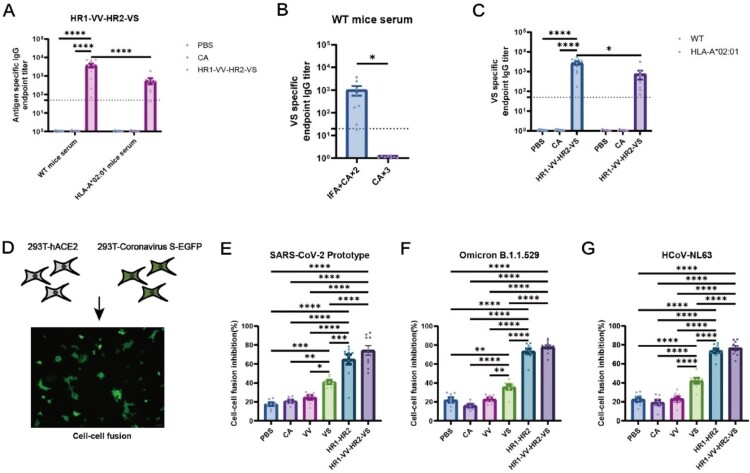
Humoral immune responses induced by different antigen vaccines in immunized mice. (A) Quantification of serum antigen-specific IgG levels by ELISA following immunization of WT and HLA-A*02:01 transgenic mice with HR1-VV-HR2-VS antigens or a control adjuvanted with CpG/Al(OH)_3_. (B) VS-specific IgG levels in sera from WT mice immunized with VS antigen adjuvanted with either CpG/IFA or CpG/Al(OH)₃ were measured by ELISA. (C) VS-specific IgG levels in sera were measured by ELISA in WT and HLA-A*02:01 transgenic mice following immunization with HR1-VV-HR2-VS antigen or a control, both adjuvanted with CpG/Al(OH)₃. (D) Fusion inhibition assay design. HEK293 T cells dual-transfected with SARS-CoV-2 S protein and eGFP reporters (effector cells) were co-cultured with HEK293T-hACE2 target cells. Sera from immunized WT mice were introduced to assess neutralizing antibody-mediated fusion blockade, which was quantified by counting eGFP + syncytia after 6 h of incubation. (E-G) Statistical analysis of serum antibody-mediated inhibition of cell fusion induced by spike proteins from SARS-CoV-2 prototype strain (E), Omicron B.1.1.529 (F), and NL63 (G) with cell surface hACE2 protein. PBS-, CA-, VV long peptide-, and VS long peptide-immunized mice (n = 8 per group); HR1-HR2 – and HR1-VV-HR2-VS-immunized mice (n = 10 per group). The dotted line represents the detection threshold in A-C. The data were analysed by two-way ANOVA (A and C), Student’s t tests (B) and one-way ANOVA (E-G). *p < 0.05, **p < 0.01, ***p < 0.001, ****p < 0.0001.

Humoral immunity analysis of immunized mouse sera revealed that both the HR1-VV-HR2-VS and HR1-HR2 vaccine formulations induced significantly higher antigen-specific antibody titres in WT mice compared to HLA-A*02:01 transgenic mice (Figure 6(A), Figure S6A). Unexpectedly, VS peptide immunization alone failed to induce detectable antibodies by ELISA. However, IFA-adjuvanted VS peptides (immunization schedule in Figure S4A-group 7) showed low but measurable VS-specific IgG titres (Figure 6(B)), suggesting the suboptimal immunogenicity of standalone peptides necessitated adjuvant optimization. Moreover, the HR1-VV-HR2-VS fusion protein demonstrated superior immunogenicity, inducing 6.8-fold (WT) and 1.3-fold (transgenic) higher VS-specific antibody titres than standalone VS peptide (Figure 6(C)). Sera from mice immunized with VS (*p* = 0.002), HR1-HR2 (*p* < 0.0001), or HR1-VV-HR2-VS (*p* < 0.0001) antigens exhibited significant neutralization activity against the SARS-CoV-2 prototype pseudovirus relative to the adjuvant-only control group (Figure S6B). While sera from mice immunized with the VS peptide or HR1-HR2 showed reduced neutralization against Omicron B.1.1.529, the HR1-VV-HR2-VS group maintained comparable potency (*p* = 0.0025 relative to the adjuvant-only group) (Figure S6C). These results suggested that the HR1-VV-HR2-VS antigen may induce neutralizing antibodies with broader cross-reactive activity. Expression of coronavirus S protein on 293 T cell membranes induced syncytia formation through specific binding with ACE2 expressed on adjacent 293 T cells (Figure 6D). The sera of HR1-VV-HR2-VS group showed 73 ± 15% fusion inhibition against SARS-CoV-2 prototype (vs 21 ± 5% for CA, *p* < 0.0001), 78 ± 6% for Omicron B.1.1.529 (vs 16 ± 6% for CA, *p* < 0.0001), and 77 ± 7% for HCoV-NL63 (vs 20 ± 8% for CA, *p* < 0.0001) (Figures 6E-G), indicating broad fusion blockade. Serum from HR1-VV-HR2-VS-immunized wild-type mice exhibited a 1.83-fold increase in antibody-dependent cellular cytotoxicity (ADCC; *p* = 0.014) and 1.40-fold increase in antibody-dependent cellular phagocytosis (ADCP; *p* = 0.139) compared to the adjuvant-only group (Figure S6D-E). Consistent with the germinal centre responses, the fusion-inhibitory activity of serum antibodies was predominantly mediated by the HR1-HR2 scaffold protein, with the VS long peptide contributing an additional enhancement.

### The HR1-VV-HR2-VS vaccine conferred superior protection against live coronavirus challenge in mice

**Figure 7.**
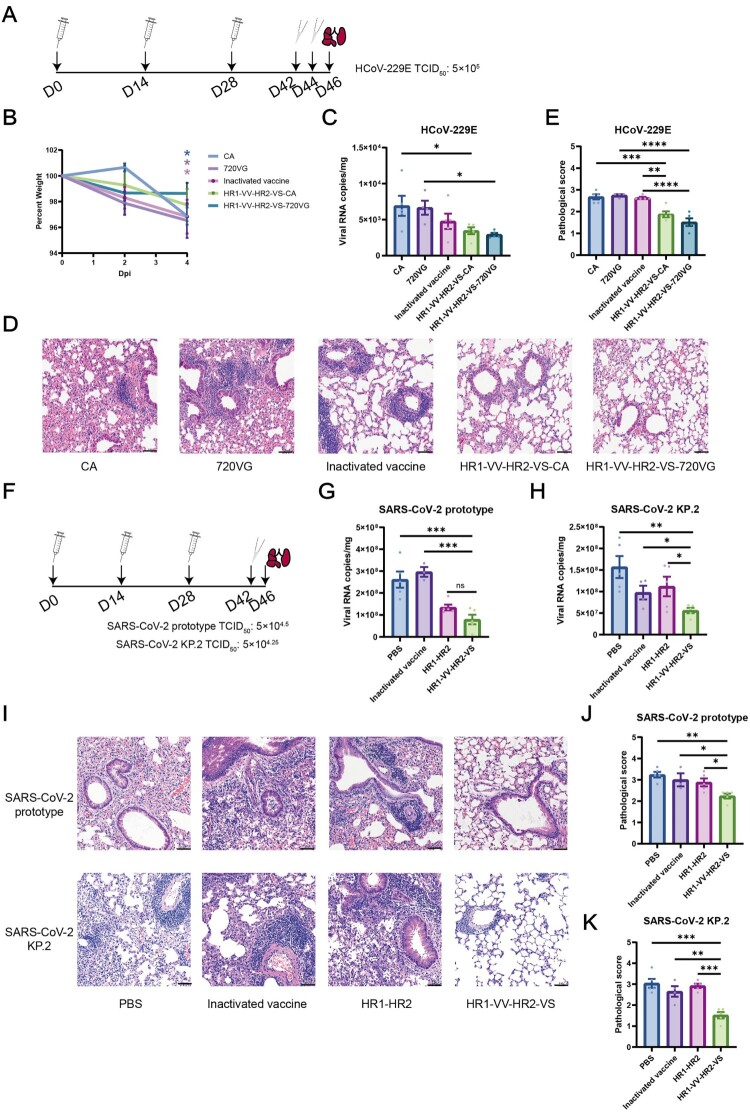
HR1-VV-HR2-VS vaccine formulation demonstrated broad protective efficacy against live coronavirus challenges. (A) Immunization and challenge strategies for BALB/c mice. BALB/c mice were intramuscularly immunized with three doses of vaccine at day 0, 14 and 28. Fourteen days after the final immunization, the animals received two sequential intranasal challenges with HCoV-229E (5 × 10⁵ TCID₅₀ per dose, 24 h between challenges). (B) Body weight measurements were recorded at day 0, 2, and 4 post infection (dpi). (C) Viral loads in the lung homogenates supernatants were quantified via qRT-PCR at 4 dpi. (D) Representative histopathological images of lung sections at 4 dpi (scale bar = 50 μm). (E) Histopathological score of lung tissues at 4 dpi. The pathological evaluation criteria encompass inflammatory cell infiltration, hemorrhage, epithelial cell injury, protein accumulation, and parenchymal wall thickening. 720VG-immunized mice (n = 4); CA-, inactivated-, HR1-VV-HR2-VS-CA – and HR1-VV-HR2-VS-720VG immunized mice (n = 5 per group). CA: CpG/Al(OH)_3_ adjuvants, 720VG: Montanide™ ISA 720 VG adjuvant. (F) Immunization and challenge strategies for K18-hACE2 mice. K18-hACE2 transgenic mice were intramuscularly immunized with three doses of vaccine at day 0, 14 and 28. 14 days after the final immunization (n = 5 per group), the animals received intranasal challenges with SARS-CoV-2 prototype (5 × 10^4.5^ TCID₅₀) or SARS-CoV-2 KP.2 (5 × 10^4.25^ TCID₅₀). (G-H) Viral loads in lung homogenate supernatants were quantified by qRT-PCR at 4 dpi with either prototype SARS-CoV-2 (G) or the KP.2 variant (H). (I) Representative histopathological images of lung sections at 4 dpi (scale bar = 50 μm). (J-K) Histopathological score of lung tissues at 4 dpi. In the prototype. The data were analysed by two-way ANOVA (B) and one-way ANOVA (C, E, G, H, J and K). Non-significant differences were labeled as “ns”. *p < 0.05, **p < 0.01, ***p < 0.001, ****p < 0.0001.

Given the limited adjuvant efficacy of the CpG/Al(OH)₃ combination, we further introduced Montanide ISA 720VG (a clinically safer alternative to IFA) and evaluated its protective immunogenicity in mice. All immunizations were administered intramuscularly using ISA 720VG-adjuvanted formulations to maintain experimental consistency. Mice immunized with HR1-VV-HR2-VS were intranasally challenged with HCoV-229E using a dual-inoculation protocol (Figures 7(A), S7A).[54] HR1-VV-HR2-VS immunization prevented significant weight loss (Figure 7(B)), reduced pulmonary viral loads by 58–65% (Figure 7(C)), and attenuated lung pathology (including pronounced inflammatory cell infiltration and alveolar septum thickening, Figure 7(D,E)) versus adjuvant-only controls, demonstrating superior protective efficacy. Based on its superior protective efficacy, Montanide ISA 720VG was selected as the adjuvant for subsequent SARS-CoV-2 challenge studies. K18-hACE2 transgenic mice were primed with inactivated vaccine and boosted twice with candidate vaccines at 2-week intervals (Figure S7B). Two weeks post-immunization, mice were intranasally challenged with SARS-CoV-2 prototype (Wuhan-Hu-1) or KP.2 variant, with viral loads and pathology assessed 4 days post-infection (Figure 7(F)). HR1-VV-HR2-VS booster immunization demonstrated superior protection, reducing lung viral loads by 76% and 79% against ancestral SARS-CoV-2, and 64% and 36% against KP.2 variant compared to PBS and inactivated vaccine controls, respectively (Figure 7(G,H)). Single-dose inactivated vaccine reduced KP.2 viral loads by 43% but showed no prototype protection, while HR1-HR2 immunization demonstrated 47% and 31% reductions against wild-type and KP.2 strains, respectively (Figure 7(G,H)). HR1-VV-HR2-VS vaccination significantly attenuated pulmonary pathology, reducing inflammatory cell infiltration and preserving alveolar structure versus controls (Figure 7(I,K)). Collectively, these data indicated that HR1-VV-HR2-VS booster immunization conferred broad protection against multiple coronaviruses, including ancestral SARS-CoV-2, the KP.2 variant, and heterologous HCoV-229E.

## Discussion

Comparative analysis of spike proteins from seven human coronaviruses and SARS-CoV-2 variants, we identified two conserved and immunodominant epitopes: VV (T-cell) and VS (B-cell), validated by COVID-19 convalescent PBMCs. Subsequently, we engineered an HR1-VV-HR2-VS fusion protein by leveraging the natural HR1-HR2 trimerization mechanism of SARS-CoV-2. Immunization with this construct enhanced both T-cell responses and VS-specific humoral immunity compared to individual peptides, while demonstrating cross-protective efficacy against ancestral SARS-CoV-2, KP.2 variant, and HCoV-229E challenges in mice.

Epitope-focused vaccines targeting conserved T/B-cell epitopes show promise for developing broadly protective coronavirus vaccines. Clinical trial results of CoVac-1 showed its ability to induce sustained, variant-resistant IFN-γ responses and robust T-cell immunity even in B-cell-deficient patients [55, 56]. Our epitope-centric vaccine design leverages evolutionarily constrained S protein regions to generate durable, cross-reactive immunity through VV/VS long peptides containing promiscuous CD8^+^ T-cell epitopes with 94% global HLA-I coverage study by Pereira Neto et al. confirmed that conserved T-cell epitope regions (including the HR1 domain and VV long peptide) within the S2 subunit significantly enhance cross-reactive T-cell recognition across Betacoronaviruses [57]. This finding provided independent support for the broad-spectrum vaccine potential of these regions.

Several HR1/HR2-based vaccine candidates have shown promising immunogenicity and protective efficacy. For instance, HR121 exhibited robust efficacy in multiple animal models [47]^,^while HR1LS elicited broad-spectrum neutralizing antibodies [50]. Our data disclosed that HR1-HR2 immunization provided limited protection against KP.2 variant challenge in mice (Figure 7G), potentially attributable to key mutations (S939F, Q954H, and N969 K) within the HR1 domain of the KP.2 S protein. Comparative analysis revealed that the HR1-VV-HR2-VS fusion peptide exhibited markedly enhanced protective efficacy against Omicron KP.2 challenge compared to the HR1-HR2 construct, demonstrating both significantly lower viral loads (Figure 7G) and reduced pulmonary pathology (Figure 7H). This protective advantage suggested that the incorporated VV/VS long peptides functionally synergize with the core HR1-HR2 domains to broaden immune recognition. The observed cross-protection against heterologous HCoV-229E infection (Figures 7C-D) further substantiates this cooperative interaction. Our findings demonstrated that the HR1-VV-HR2-VS fusion protein exhibited significantly enhanced and broader protective efficacy compared to constructs containing only the HR1 and HR2 domains. Our study established an innovative fusion of conserved T/B-cell epitopes with the HR1-HR2 scaffold as a novel vaccine antigen. This design successfully elicits concurrent humoral and cellular immunity, marking a conceptual transition from epitope mapping to functional antigen design for developing broad-spectrum peptide vaccines.

COVID-19 patient sera demonstrated cross-reactivity against heterologous HCoV S2 domains, supporting its potential as a target for pancoronavirus vaccines [58]. However, the neutralizing antibody responses elicited by the S2 subunit is limited, reflected in the incomplete viral clearance (Figure 7) and modest neutralizing antibody titres (Figure S6) observed with HR1-VV-HR2-VS immunization. Consequently, S2-targeted vaccines are highly dependent on adjuvant selection for optimal immunogenicity. The CF501 adjuvant was shown to induce higher neutralizing antibody titres than alum in HR1LS vaccination [19], consistent with our findings that Freund's adjuvant enhanced VS peptide-specific humoral immunity (Figure 6B). Future work will optimize this platform through adjuvant and delivery system evaluations.

We observed distinct T-cell responses to peptides SP2-4 and SP10-12 in wild-type versus HLA-A*02:01 transgenic mice. These differences may involve HLA restriction and binding affinity variations. For example, the SP4 epitope does not bind HLA-A*02:01. Additionally, epitopes in SP3, SP10, and SP13 are predicted to bind weakly. Another contributing factor could be that the stimulating peptides do not match the peptides naturally processed by antigen-presenting cells. This mismatch may account for the lack of significant responses to the HLA-A*02:01-binding epitopes in SP2 and SP12. In addition, HLA-A*02:01-transgenic mice exhibited reduced germinal centre reactions and humoral responses versus WT controls (Figure 6A), likely due to human β−2 microglobulin substitution impairing FcRn-mediated IgG protection [59]. This resulted in accelerated IgG catabolism and significantly lower antigen-specific antibody titres compared to WT mice at equivalent timepoint. In addition, VS peptide immunization significantly reduced Tfh cell frequencies versus controls in wild-type mice (Figure 5B), potentially mediated by IL-2-mediated suppression of Tfh differentiation [60]. Consistent with this mechanism, both VS long peptide and peptide pools potently stimulated IL-2 production in human PBMCs (Figure 2A-B).

Nevertheless, this study has two main limitations, incomplete viral clearance was observed despite significant viral load reduction, and mucosal immunity was not evaluated. Future work will optimize adjuvant formulations, particularly TLR agonists and STING activators, to enhance both systemic and mucosal protection.

Taken together, our study introduced a rational vaccine design that was founded on two synergistic components: the strategic incorporation of conserved and efficient T- and B-cell epitopes, and an HR1/HR2 scaffold that played a dual role. This scaffold protein not only robustly enhanced the immunogenicity of these epitopes but also concurrently provided independent protective immunity. This strategy effectively transcended the limitations of minimal epitope vaccines and established a versatile platform for broad-spectrum protection.

## Supplementary Material

Supplementary Figure and legends.docx
