# Supplementary material for: Receptor binding domain-independent pancoronavirus vaccine design by fusion of conserved T/B Epitopes
Source: Emerg Microbes Infect. 2026 Feb 11;15(1):2631206. doi: 10.1080/22221751.2026.2631206 (PMC12927409; doi:10.1080/22221751.2026.2631206)
Supplement: Supplementary Figure and legends.docx [file TEMI_A_2631206_SM4010.docx]

**Supplementary** **figures and figure legends**

**
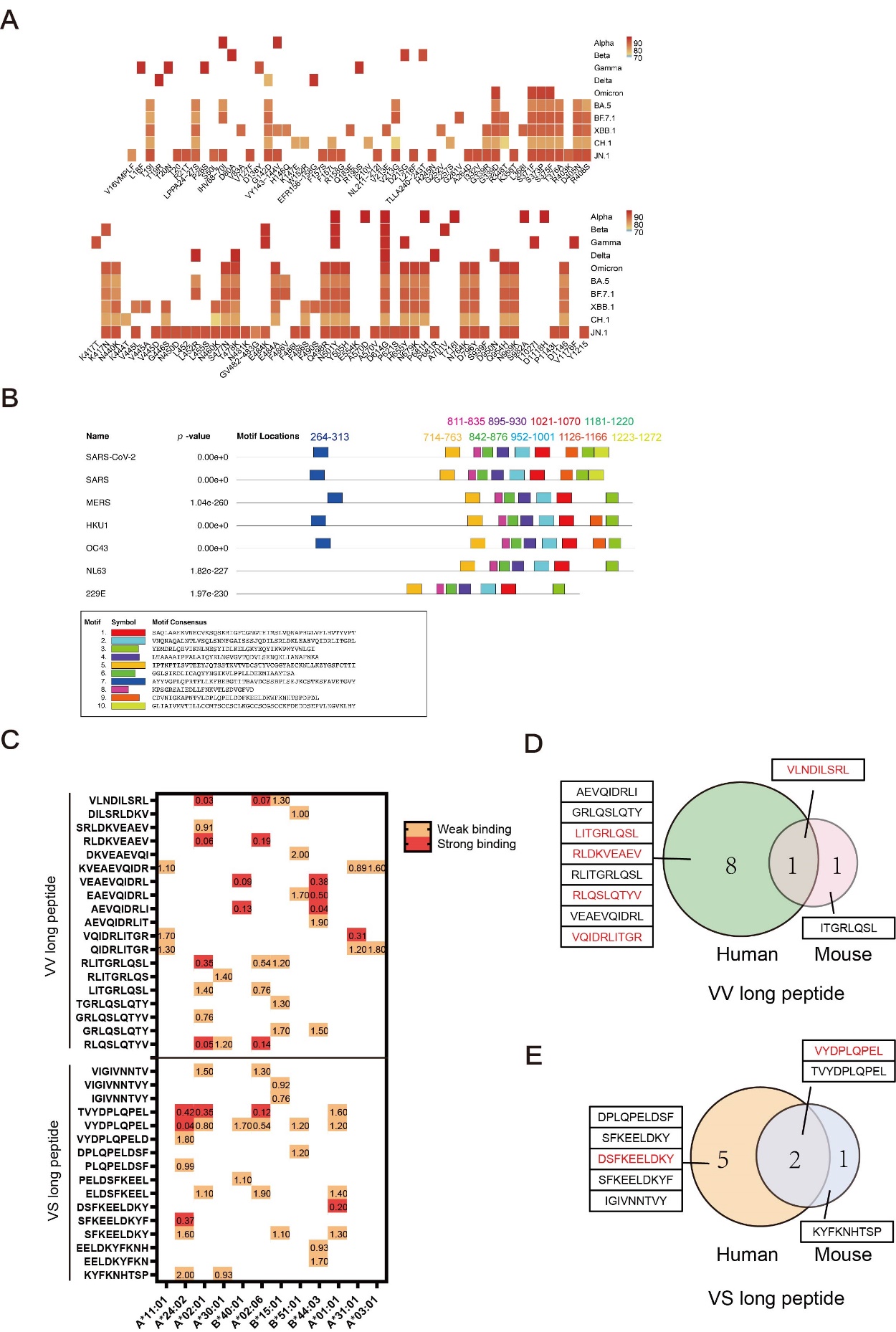
**

**Figure S1.** **Conservation analysis of the spike protein across SARS-CoV-2 variants and human-infecting coronaviruses.** A) Mutation profiles of prevalent SARS-CoV-2 variants. The x-axis indicates amino acid mutation positions in the spike protein, whereas the y-axis shows different SARS-CoV-2 variants. The color gradients represent the frequency of amino acid variations across variants. Data were obtained from NGDC (<https://ngdc.cncb.ac.cn/ncov/variation/sequence/compare>). B) Top10 conserved motifs in spike proteins from seven human coronaviruses. Sequence alignment via MEME (https://meme-suite.org/meme/tools/meme) identified the 10 most conserved motifs, with SARS-CoV-2 VV and VS peptides ranking 2nd and 9th, respectively. C) Predicted binding affinity of CD8^+^ T-cell epitopes in VV and VS peptides for various HLA alleles. The x-axis displays different HLA alleles, whereas the y-axis shows the predicted CD8^+^ T-cell epitopes from the IEDB database. The color scale indicated binding strength on the basis of %rank scores (strong binding: %rank <0.5; weak binding: 0.5<%rank <2). Predictions were generated via NetMHCpan 4.1 EL (<http://tools.iedb.org/mhci/>). D-E) Venn diagrams showed overlapping CD8^+^ T-cell epitopes with strong binding affinities for the VV (D) and VS (E) peptides for human and murine MHC-I molecules. The left circle represents epitopes that strongly bind to HLA, whereas the right circle represents those that strongly bind to murine MHC-I. The overlapping region displays epitopes that strongly bind to both. Red labels identify epitopes selected for subsequent cellular experiments.

**
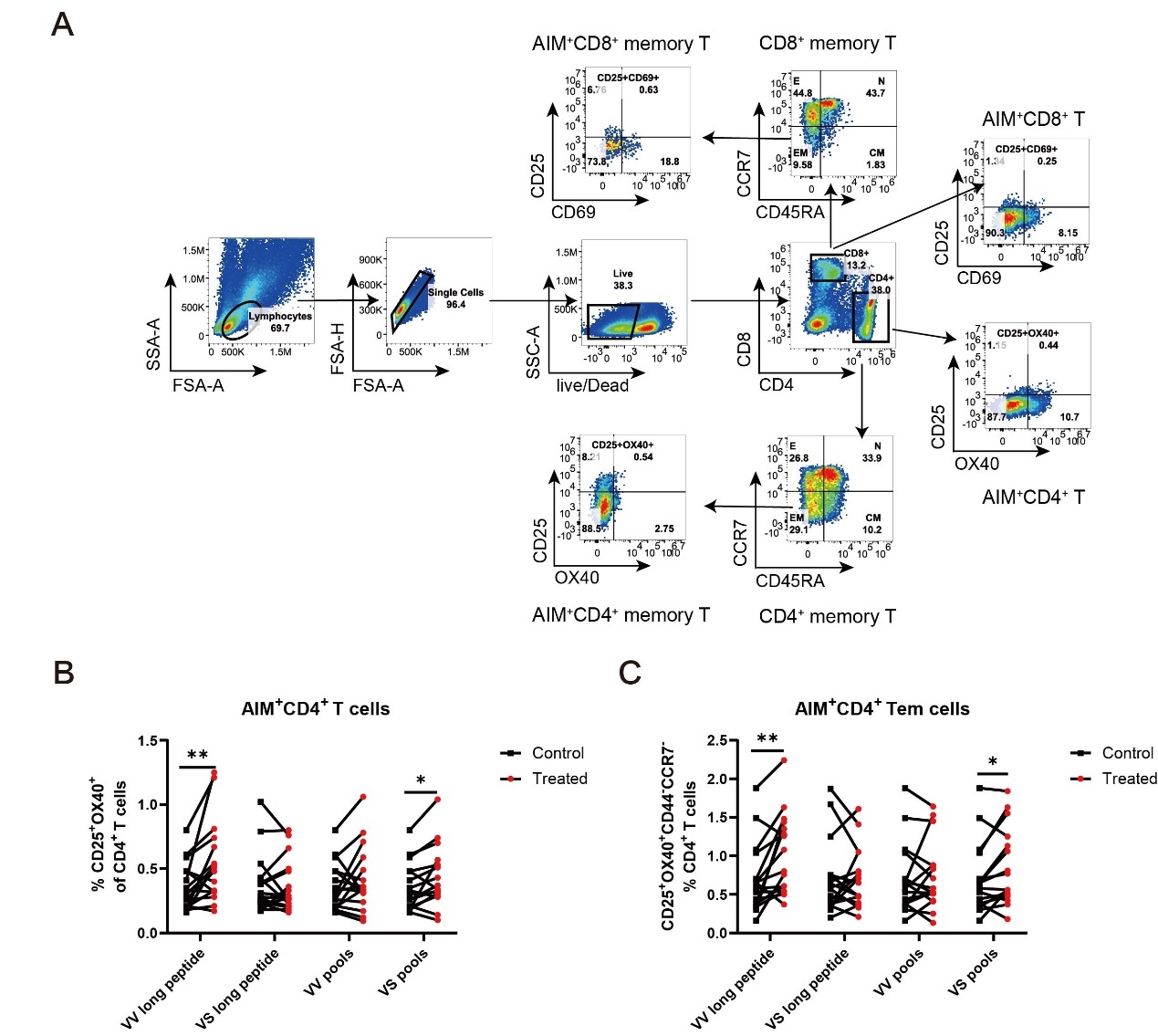
**

**Figure S2. Specific CD4^+^ T-cell immune responses of COVID-19 convalescent patients.** A) Gating strategies for flow cytometry after peptide stimulation of PBMCs. B-C) Proportion of AIM^+^CD4^+^ T cells (CD4^+^CD25^+^OX40^+^) or AIM^+^CD4^+^ Tem cells (CD4^+^CD45RA^-^CCR7^-^CD25^+^OX40^+^) in PBMCs (n=16) following 24 hours of stimulation with VV, VS, or their peptide pools. PBMCs stimulated with PBS or DMSO served as negative controls. A paired t test was used for the results. *p < 0.05, **p < 0.01.

**
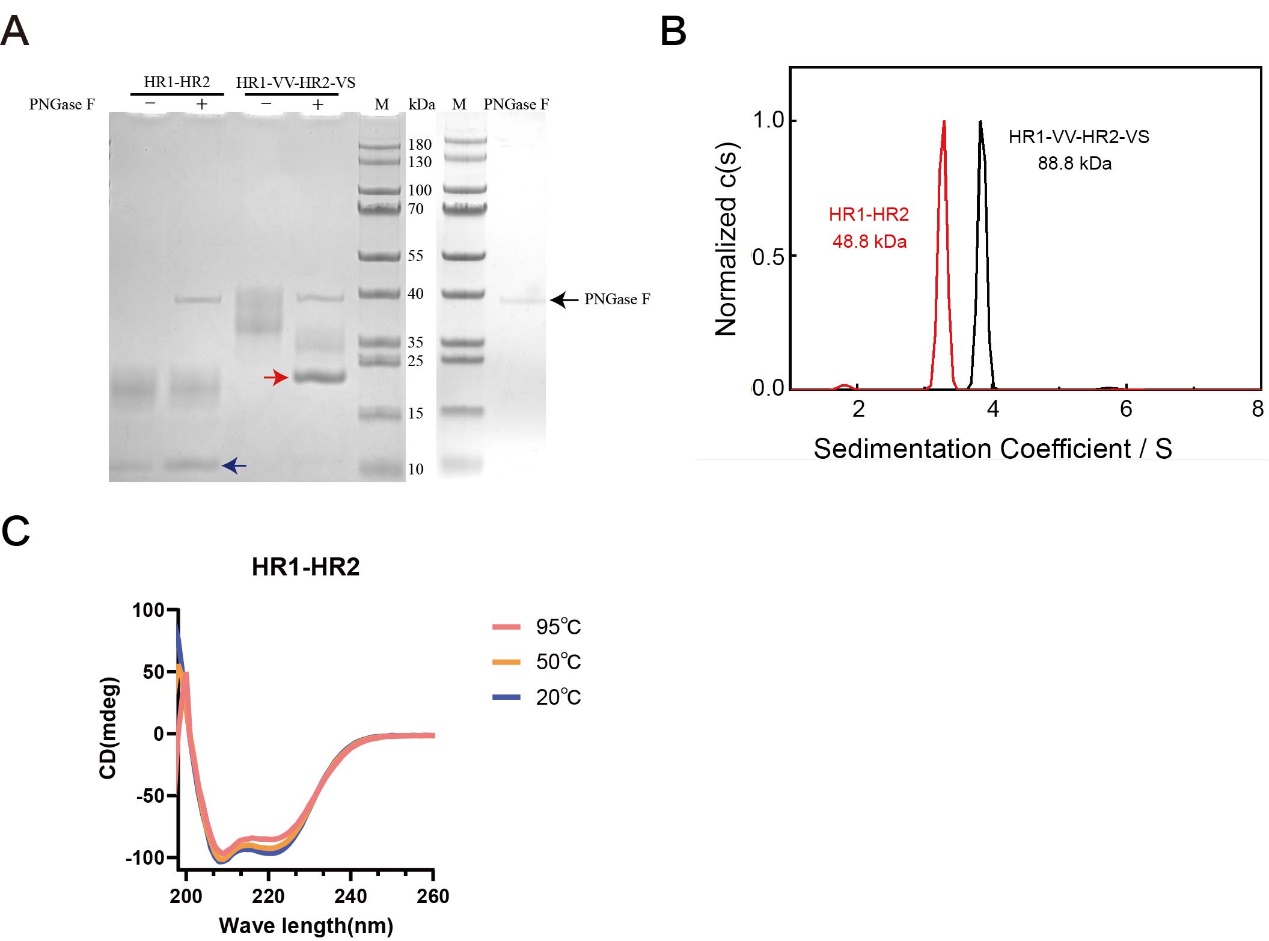
**

**Figure S3.** **Physicochemical characterization of the fusion proteins HR1-VV-HR2-VS and HR1-HR2.** A) The HR1-VV-HR2-VS and HR1-HR2 recombinant proteins were subjected to deglycosylation with PNGase F, and were further analyzed via SDS-PAGE. Black, red, and blue arrows indicate the positions of the PNGase F protease, the theoretical size of the HR1-VV-HR2-VS protein, and the theoretical size of the HR1-HR2 protein, respectively. B) Analytical ultracentrifugation analysis of HR1-HR2 (red) and HR1-VV-HR2-VS (black) fusion proteins. C) Circular dichroism spectral analysis of the HR1-HR2 protein. Scans recorded at 20°C (blue), 50°C (yellow), and 95°C (red) were shown.

**
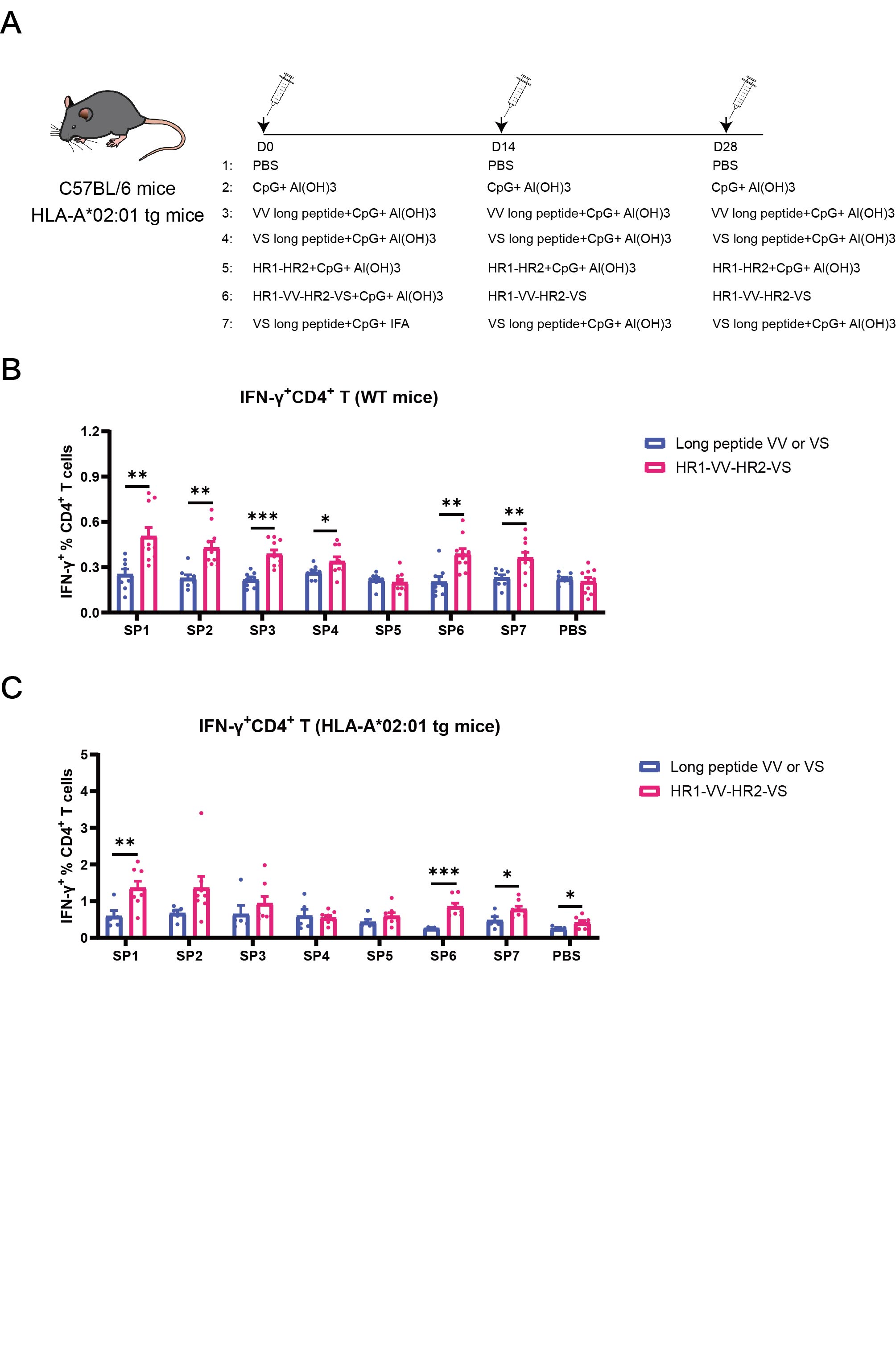
**

**Figure S4.** **Antigen-specific CD4^+^ T-cell immune responses induced by individual T-cell epitopes in mouse splenic lymphocytes.** A) Antigen immunization strategies for mice based on immune evaluation. B-C) The percentage of IFN-γ^+^CD4^+^ T cells in splenocytes from WT mice (B) and HLA-A*02:01 transgenic mice (C) following stimulation with individual CD8^+^ T-cell epitopes. The SP1-5 peptides are located within the VV long peptide segment, while SP6 and SP7 reside in the VS long peptide region. Mice cohorts included: WT mice (peptide: n=8; fusion protein: n=10) and HLA-A*02:01 transgenics (peptide: n=5; fusion protein: n=8). The data were analyzed by Student's t test by B-C. **p* < 0.05, ***p* < 0.01, ****p* < 0.001.

**
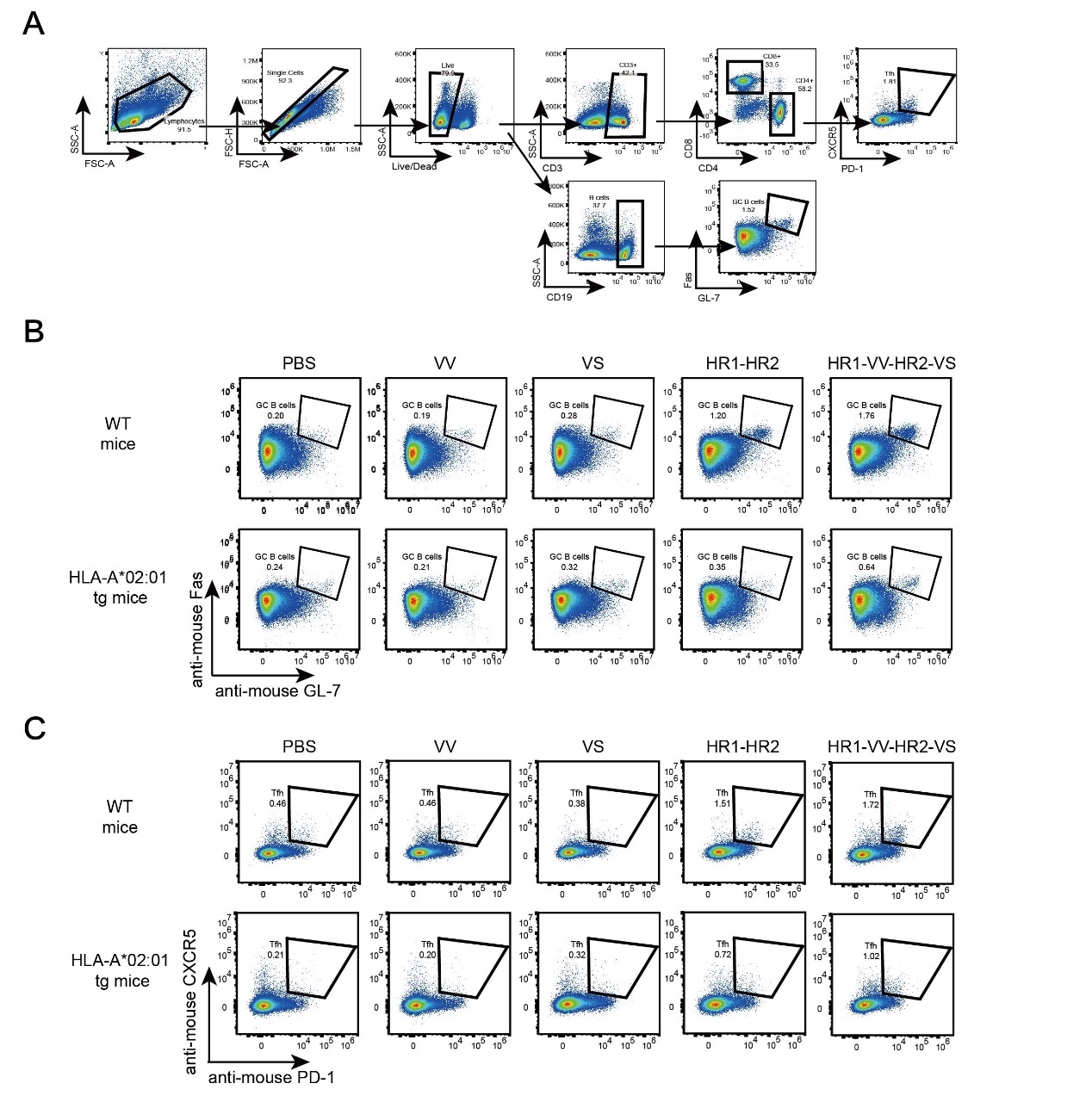
**

**Figure S5.** **Germinal center responses in splenocytes from immunized HLA-A*02:01 transgenic mice.** A) Gating strategy for GC B cells (CD19^+^GL-7^+^Fas^+^) and T follicular helper (Tfh, CD4^+^CXCR5^+^PD-1^+^) cells in mouse splenic lymphocytes. B-C) Representative flow cytometry plots of GC B (B) cells and Tfh (C) cells from immunized WT and HLA-A*02:01 transgenic mice.

**
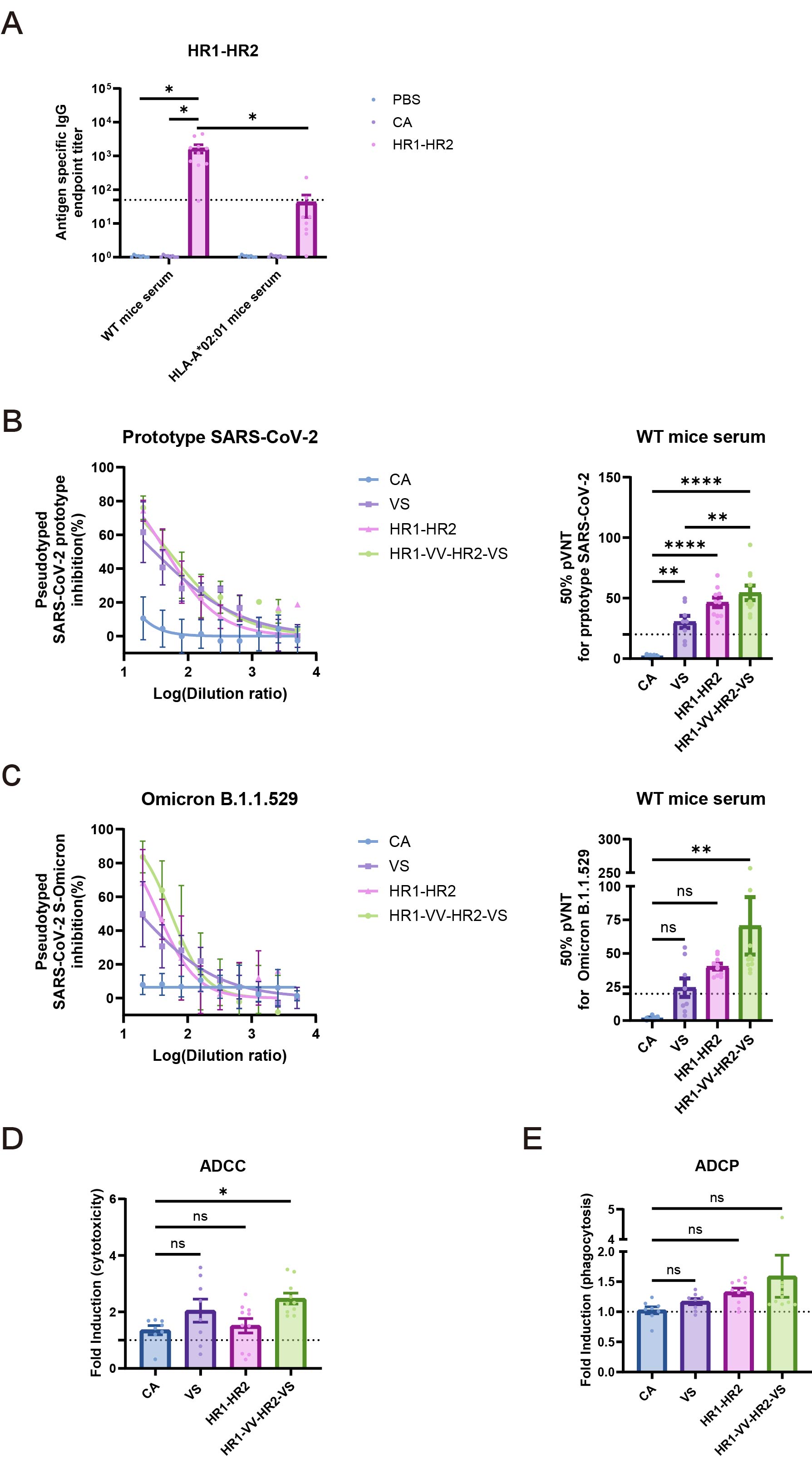
**

**Figure S6.** **Neutralizing antibody titers induced by different antigen vaccines in immunized mice.** A) Quantification of serum antigen-specific IgG levels by ELISA following immunization of WT and HLA-A*02:01 transgenic mice with HR1-HR2 antigens or a control adjuvanted with CpG/Al(OH)_3_. B-C) Neutralization profiles of serum antibodies from WT mice immunized with VS, HR1-HR2, or HR1-VV-HR2-VS antigens. (Left) Inhibition curves against pseudotyped SARS-CoV-2 (prototype strain, A) and Omicron B.1.1.529 (B) variants. (Right) Statistical comparison of neutralizing antibody titers (ID₅₀ values). D-E) Antibody-dependent cellular cytotoxicity (ADCC) activities and antibody-dependent cellular phagocytosis (ADCP) activities of serum samples that collected from each group. WT mice immunized with the PBS (n=8), CA (n=8), VS long peptide (n=8), HR1-HR2 (n=10), or HR1-VV-HR2-VS (n=10) fusion proteins. The dotted line represents the detection threshold. The data were analyzed via two-way ANOVA in A and one-way ANOVA in B-E. **p* < 0.05, ***p* < 0.01, ****p* < 0.001, *****p* < 0.0001. Non-significant differences were labeled as “ns.”

**
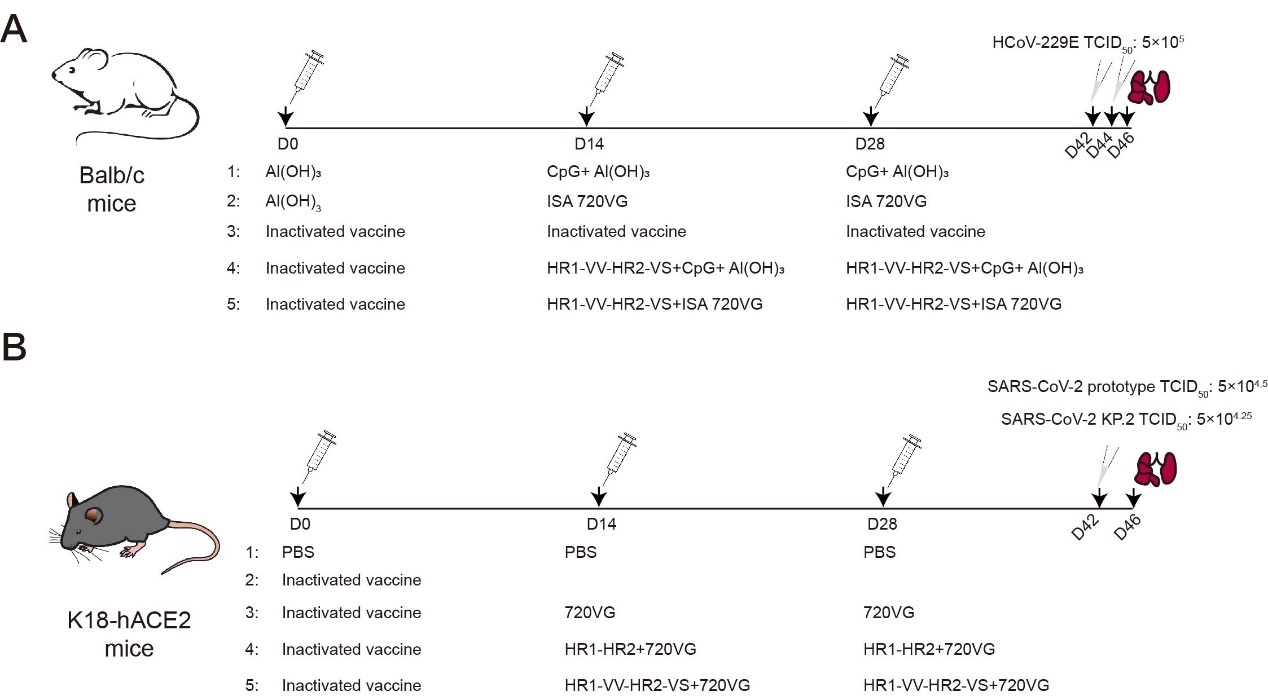
**

**Figure S7. Immunization strategies for mice before coronavirus challenge.** A) Immunization strategies for mice before HCoV-229E virus challenge. B) Immunization strategies for mice before SARS-CoV-2 prototype strain and KP.2 variant challenge.

**Table S1.** **Sequence of VV, VS and related CD8^+^ T cell epitopes.**

|  | **Region** | **Long-peptide sequence** | **Peptide name** | **Peptide** |
| --- | --- | --- | --- | --- |
| VV | S976-1008 | VLNDILSRLDKVEAEVQIDRLITGRLQSLQTYV | SP1 | VLNDILSRL |
|  |  |  | SP2 | RLDKVEAEV |
|  |  |  | SP3 | LITGRLQSL |
|  |  |  | SP4 | VQIDRLITGR |
|  |  |  | SP5 | RLQSLQTYV |
| VS | S1129-1170 | VIGIVNNTVYDPLQPELDSFKEELDKYFKNHTSPDVDLGDIS | SP6 | VYDPLQPEL |
|  |  |  | SP7 | DSFKEELDKY |

**Table S2.** **Population coverage analysis of CD8^+^ T cell epitopes in VV and VS long peptides.**

| **Population/area** | **Class I** | | |
| --- | --- | --- | --- |
|  | **Coverage^a^** | **Average_hit^b^** | **Pc90^c^** |
| World | 94.07% | 2.74 | 1.15 |
| China | 92.26% | 2.11 | 1.07 |
| Hong Kong | 96.58% | 2.35 | 1.23 |
| East Asia | 95.07% | 2.31 | 1.15 |
| Europe | 97.02% | 3.0 | 1.3 |
| North America | 94.19% | 2.89 | 1.18 |
| United States | 94.22% | 2.89 | 1.18 |
| Southeast Asia | 93.18% | 2.15 | 1.09 |
| Northeast Asia | 92.74% | 2.14 | 1.08 |
| West Indies | 92.35% | 2.63 | 1.1 |
| Oceania | 91.7% | 1.75 | 1.03 |

**Table S3.** **Table of COVID-19 vaccination and infection information for study volunteers.**

| **Donor** | **Gender** | **Age** | **Vaccination schedule** | **Infection** | **PBMCs Collection** |
| --- | --- | --- | --- | --- | --- |
| 1 | M | 27 | 1st: May 15, 2021 (Sinovac-CoronaVac COVID-19 vaccine) 2nd: June 5, 2021 (Sinovac-CoronaVac COVID-19 vaccine) 3rd: March 18, 2022 (Sinovac-CoronaVac COVID-19 vaccine) 4th: December 19, 2022 (Zifivax COVID-19 vaccine) | May 13, 2023 | May 31, 2024 |
| 2 | M | 31 | 1st: April 30, 2021 (Zifivax COVID-19 vaccine) 2nd: June 2, 2021 (Zifivax COVID-19 vaccine) 3rd: July 3, 2021(Zifivax COVID-19 vaccine) | November 7, 2022 | May 31, 2024 |
| 3 | M | 45 | 1st: May 21, 2021 (Sinovac-CoronaVac COVID-19 vaccine) 2nd: June 11, 2021 (Sinovac-CoronaVac COVID-19 vaccine) | December 16, 2022 | May 31, 2024 |
| 4 | M | 23 | 1st: May 19, 2021 (Sinovac-CoronaVac COVID-19 vaccine) 2nd: May 19, 2021 (Sinopharm COVID-19 vaccine ) 3rd: December 16, 2021 (Sinopharm COVID-19 vaccine) | December 24, 2022 | May 31, 2024 |
| 5 | F | 26 | 1. December 16, 2021 (AstraZeneca Vaxzevria)  2. September 9, 2021 (AstraZeneca Vaxzevria)  3. February 14, 2022 (Pfizer Comirnaty ) | May 22, 2022 | May 31, 2024 |
| 6 | M | 34 | 1st: May 17, 2021 (Sinovac-CoronaVac COVID-19 vaccine) 2nd: June 7, 2021 (Sinovac-CoronaVac COVID-19 vaccine) 3rd: January 28, 2022 (Sinovac-CoronaVac COVID-19 vaccine) | December 28, 2022 | May 31, 2024 |
| 7 | M | 35 | 1st: April 21, 2021 (Sinovac-CoronaVac COVID-19 vaccine) 2nd: May 20, 2021 (Sinovac-CoronaVac COVID-19 vaccine) 3rd: December 9, 2021(Sinovac-CoronaVac COVID-19 vaccine) | December 25, 2022 | May 31, 2024 |
| 8 | F | 29 | 1st: December 9, 2021 (Sinovac-CoronaVac COVID-19 vaccine) 2nd: June 8, 2021 (Sinovac-CoronaVac COVID-19 vaccine) 3rd: February 21, 2022 (Sinovac-CoronaVac COVID-19 vaccine) | December 23, 2022 | May 31, 2024 |
| 9 | F | 30 | 1st: June 4, 2021 (Sinovac-CoronaVac COVID-19 vaccine) 2nd: June 28, 2021 (Sinopharm COVID-19 vaccine （Beijing）) | May, 2023 | June 26, 2024 |
| 10 | F | 27 | 1st: April 21, 2021 (Sinovac-CoronaVac COVID-19 vaccine) 2nd: May 20, 2021 (Sinovac-CoronaVac COVID-19 vaccine) 3rd: December 9, 2021 (Sinovac-CoronaVac COVID-19 vaccine) | December 25, 2022 | June 26, 2024 |
| 11 | M | 34 | 1st: June 9, 2021 (Zifivax COVID-19 vaccine) 2nd: June 9, 2021 (Zifivax COVID-19 vaccine) 3rd: August 8, 2021 (Zifivax COVID-19 vaccine) | January, 2023 | June 26, 2024 |
| 12 | F | 26 | 1st: April 17, 2021 (Zifivax COVID-19 vaccine) 2nd: May 18, 2021 (Zifivax COVID-19 vaccine) 3rd: June 19, 2021 (Zifivax COVID-19 vaccine) | September, 2023 | June 26, 2024 |
| 13 | M | 25 | 1st: May 15, 2021 (Sinovac-CoronaVac COVID-19 vaccine) 2nd: May 15, 2021 (Sinovac-CoronaVac COVID-19 vaccine) 3rd: March 19, 2022 (Zifivax COVID-19 vaccine) | December, 2022 | June 26, 2024 |
| 14 | M |  | 1st: April 21, 2021 (Sinovac-CoronaVac COVID-19 vaccine) 2nd: April 21, 2021 (Sinopharm COVID-19 vaccine (Beijing)) | December, 2022 | June 26, 2024 |
| 15 | F | 26 | 1st: May 8, 2021 (Zifivax COVID-19 vaccine) 2nd: May 8, 2021 (Zifivax COVID-19 vaccine) 3rd: July 9, 2021 (Zifivax COVID-19 vaccine) | December 16, 2022 | June 26, 2024 |
| 16 | M | 38 | 1st: July, 2021 (Sinovac-CoronaVac COVID-19 vaccine) 2nd: August, 2021 (Sinovac-CoronaVac COVID-19 vaccine) | December 17, 2022 | 2024/6/26 |

**Table S4.** **HR1 and HR2-associated T cell epitope sequences.**

|  | **Name** | **Sequence** | **Region** |
| --- | --- | --- | --- |
| HR1 | SP8 | VTQNVLYENQKLIANQF | S911-927 |
|  | SP9 | ANQFNSAIGKIQDSL | S924-938 |
|  | SP10 | SSTASALGKLQDVVN | S939-953 |
| HR2 | SP11 | KEIDRLNEVAKNLNESL | S1181-1197 |
|  | SP12 | LNEVAKNLNESLIDLQELGK | S1186-1205 |
|  | SP13 | DISGINASVVNIQKEIDR | S1168-1185 |
